# Editorial: Leveraging machine learning for omics-driven biomarker discovery

**DOI:** 10.3389/fmolb.2022.1119644

**Published:** 2023-01-09

**Authors:** Sheng Li, Charles Hsu, Tianyi Zhao, Liangcan He

**Affiliations:** ^1^ Zhongnan Hospital, Wuhan University, Wuhan, China; ^2^ Department of Population Medicine, College of Medicine, Qatar University, Doha, Qatar; ^3^ Harbin Institute of Technology, Harbin, China

**Keywords:** biomarker, machine learning, cancer, omics data analysis, computational resource

Here, we organized a Research Topic on “Leveraging Machine Learning for Omics-driven Biomarker Discovery.” In total, about 12 outstanding works were presented in this thematic issue, and they have been highlighted as follows.

• Chen et al. comprehensively investigated the expression dysregulation and prognostic significance of HSF2, and the relationship with clinicopathological parameters and immune infiltration across cancers. Their study revealed the varied expression of HSF2 in different types and stages of cancers, which suggests that the effects of HSF2 on oncogenesis may vary across different cancer types. A significant correlation between HSF2 expression and patients’ prognosis was observed. HSF2 expression was strongly related to immune cell infiltration, immune checkpoints, TMB, and MSI. They integrated existing data to explore the potential function of HSF2 in cancers and provides insights for targeting HSF2 to improve the therapeutic efficacy of immunotherapy.
• Zhang et al. found that CANX, BID, NAMPT, and BIRC5 were immune-autophagy-related genes with independent prognostic value, and the risk prognostic model based on them was theyll constructed. Through GSE168845, immune-related genes, autophagy-related genes, and immune-autophagy-related differentially expressed genes (IAR-DEGs) were identified. Then, the lasso Cox regression model was established to evaluate the correlation of IAR-DEGs with the immune score, immune checkpoints, methylation, and one-class logistic regression (OCLR) score. Further analysis showed that CANX, BID, NAMPT, and BIRC5 were potential targets and effective prognostic biomarkers for immunotherapy combined with autophagy in kidney renal clear cell carcinoma.
• Sun et al. analyzed the correlation of hub mIR-DEGs with clinicopathological factors, immune invasion, and immune checkpoints, and re-evaluated the expression of hub mIR-DEGs and their effect on the tumor by OCLR scores in KIRC. Co-expressed metastatic immune-related differentially expressed genes (mIR-DEGs) were screened out, and the mIR-DEGs-based prognostic model that had good predictive potential was established. In addition, targeted small-molecule drugs were predicted for mIR-DEGs. This study preliminarily confirmed that FGF17, PRKCG, SSTR1, and SCTR were targeted genes that can be used as potential therapeutic targets and prognostic biomarkers for renal cancer. Preliminary validation found that PRKCG and SSTR1 were consistent with predictions.
• Zhong et al. objectives are to screen for characteristic genes specific to PTC and establish an accurate model for diagnosis and prognostic evaluation of PTC. They screened differentially expressed genes in TCGA database and discovered a three-gene signature (GJB4, RIPPLY3, ADRA1B) that was statistically significant and externally validated. For experimental validation, immunohistochemistry in tissue microarrays showed that thyroid samples’ proteins expressed by this three-gene were differentially expressed. The protocol discovered a robust three-gene signature that can distinguish prognosis, which will have daily clinical application.
• Chen et al. proposed a method based on Gradient Boosting Decision Tree (GBDT) to identify the susceptible genes of gastric cancer through a gene interaction network. Based on the known genes related to gastric cancer, they collected more genes that can interact with them and constructed a gene interaction network. Random Walk was used to extract the network association of each gene and they used GBDT to identify the gastric cancer-related genes. To verify the AUC and AUPR of their algorithm, they implemented 10-fold cross-validation. GBDT achieved AUC of .89 and AUPR of .81. This work selected ftheir other methods to compare with GBDT and found GBDT performed best.
• Zhou et al. aimed to satisfy the increasing demand for novel sensitive biomarkers and potential therapeutic targets in the treatment of GII and GIII gliomas. Their study revealed the multi-omics landscape of H2BC12 in gliomas through bioinformatics approaches. They identified the differentially up-regulated expression of H2BC12 in GII and GIII glioma tissue and proved its significant ability in predicting the adverse overall survival of GII and GIII gliomas patients. They verified that H2BC12 was a promising biomarker for the diagnosis and prognosis of patients with WHO grade II and III gliomas In a forward-looking way.
• Xia et al. purposed Xgboost to identify RP-related genes. Xgboost adds a regular term to control the complexity of the model, hence using Xgboost to find out true RD-related genes from complex and massive genes is suitable. The problem of overfitting can be avoided to some extent. To verify the potheyr of Xgboost to identify RD-related genes, they did 10-cross validation and compared it with three traditional methods: Random Forest, Back Propagation network, and Support Vector Machine. The accuracy of Xgboost is 99.13% and AUC is much higher than the other three methods. Therefore, this article can provide technical support for the efficient identification of RD-related genes and help researchers have a deeper understanding of the genetic characteristics of RD.
• Xiao et al. identified familial cohorts showing MMD susceptibility and performed THEYS on five affected individuals to identify susceptibility loci, which identified point mutation sites in the titin (TTN) gene. Moreover, TTN mutations were not found in a cohort of 50 sporadic MMD cases. They also analyzed mutation frequencies and used bioinformatic predictions to reveal mutation harmfulness, functions, and probabilities of disease correlation. rs771533925 and rs72677250 were likely harmful mutations with the involvement of TTN in MMD etiology-related pathways. CRISPR-Cas12a assays designed to detect TTN mutations provided results consistent with THEYS analysis, which was further confirmed by Sanger sequencing. This study recognized TTN as a new familial gene marker for moyamoya disease and demonstrated that CRISPR-Cas12a has the advantages of rapid detection, low cost, and simple operation, and has broad prospects in the practical application of rapid detection of MMD mutation sites.
• Fan et al. explored the pharmacological mechanisms of Chongcaoyishen decoction (CCYSD) against chronic kidney disease (CKD) *via* network pharmacology analysis combined with experimental validation. The bioactive components and potential regulatory targets of CCYSD were extracted from the TCMSP database, and the putative CKD-related target proteins were collected from the GeneCards and OMIM database. 114 kinds of cellular functional activities and 112 related cellular signaling pathways were involved in this network pharmacological analysis. Except for the autophagy and oxidative stress injury, the mechanism of CCYSD against CKD may also relate to inflammatory injury, cell cycle regulation, apoptosis, and other mechanisms. Their work provided an integrative network pharmacology approach combined with *in vivo* experiments to explore underlying mechanisms governing the CCYSD, promoting the explanation and understanding of CCYSD in CKD’s treatment.
• Chen et al. aimed to illustrate what topics the research focused on and how they varied in different periods of all the studies on brain metastases with topic modeling. They used the latent Dirichlet allocation model to analyze the titles and abstracts of 50,176 articles on brain metastases retrieved from web of Science, Embase, and MEDLINE. The work further stratified the articles to find out the topic trends of different periods. The study identified that a rising number of studies on brain metastases were published in recent decades at a higher rate than all cancer articles. Overall, the major themes focused on treatment and histopathology. Radiotherapy took over the first and third places in the top 20 topics. Since the 2010s, increasing attention concerned with gene mutations. Targeted therapy was a popular topic of brain metastases research after 2020.
• Yi et al. found candidate prognostic biomarkers and provided clinicians with an accurate method for survival prediction of ACC *via* bioinformatics methods. Linear discriminant analysis, K-nearest neighbor, support vector machine, and time-dependent ROC were performed to identify meaningful prognostic biomarkers (MPBs). Four MPBs (ASPM, BIRC5, CCNB2, and CDK1) with high accuracy of survival prediction were screened out, and their mutations and copy number variants were associated with the overall survival of ACC patients. They established two nomograms which provided clinicians with an accurate, quick, and visualized method for survival prediction, which might constitute a breakthrough in the treatment and prognosis prediction of patients with ACC.
• Li et al. aimed to investigate if machine learning approaches can be used to predict postoperative unplanned 30-day hospital readmission in old surgical patients. They extracted demographic, comorbidity, laboratory, surgical, and medication data of elderly patients older than 65 who underwent surgeries under general anesthesia in west China Hospital, Sichuan University from July 2019 to February 2021. Different machine learning approaches were performed to evaluate whether unplanned 30-day hospital readmission can be predicted. Model performance was assessed using the following metrics: AUC, accuracy, precision, recall, and F1 score; and RF + XGBoost showed the best prediction capability. The most five important features of RF + XGBoost were operation duration, white blood cell count, BMI, total bilirubin concentration, and blood glucose concentration. Machine learning algorithms can accurately predict postoperative unplanned 30-day readmission in elderly surgical patients.

